# Item development process and analysis of 50 case-based items for implementation on the Korean Nursing Licensing Examination

**DOI:** 10.3352/jeehp.2017.14.20

**Published:** 2017-09-11

**Authors:** In Sook Park, Yeon Ok Suh, Hae Sook Park, So Young Kang, Kwang Sung Kim, Gyung Hee Kim, Yeon-Hee Choi, Hyun-Ju Kim

**Affiliations:** 1 College of Nursing, Chungnam National University, Daejeon, Korea; 2 Department of Nursing, Soonchunhyang University, Cheonan, Korea; 3 Department of Nursing, Dongyang University, Yeongju, Korea; 4 College of Nursing, Catholic University of Pusan, Busan, Korea; 5 Catholic Comprehensive Cancer Institute, Seoul Saint Mary’s Hospital, Catholic University of Korea, Seoul, Korea; 6 Seoul Women’s College of Nursing, Seoul, Korea; 7 College of Nursing, Kyungpook National University, Daegu, Korea; Hallym University, Korea

**Keywords:** Case studies, Koreans, Nursing, Licensing

## Abstract

**Purpose:**

The purpose of this study was to improve the quality of items on the Korean Nursing Licensing Examination by developing and evaluating case-based items that reflect integrated nursing knowledge.

**Methods:**

We conducted a cross-sectional observational study to develop new case-based items. The methods for developing test items included expert workshops, brainstorming, and verification of content validity. After a mock examination of undergraduate nursing students using the newly developed case-based items, we evaluated the appropriateness of the items through classical test theory and item response theory.

**Results:**

A total of 50 case-based items were developed for the mock examination, and content validity was evaluated. The question items integrated 34 discrete elements of integrated nursing knowledge. The mock examination was taken by 741 baccalaureate students in their fourth year of study at 13 universities. Their average score on the mock examination was 57.4, and the examination showed a reliability of 0.40. According to classical test theory, the average level of item difficulty of the items was 57.4% (80%–100% for 12 items; 60%–80% for 13 items; and less than 60% for 25 items). The mean discrimination index was 0.19, and was above 0.30 for 11 items and 0.20 to 0.29 for 15 items. According to item response theory, the item discrimination parameter (in the logistic model) was none for 10 items (0.00), very low for 20 items (0.01 to 0.34), low for 12 items (0.35 to 0.64), moderate for 6 items (0.65 to 1.34), high for 1 item (1.35 to 1.69), and very high for 1 item (above 1.70). The item difficulty was very easy for 24 items (below −2.0), easy for 8 items (−2.0 to −0.5), medium for 6 items (−0.5 to 0.5), hard for 3 items (0.5 to 2.0), and very hard for 9 items (2.0 or above). The goodness-of-fit test in terms of the 2-parameter item response model between the range of 2.0 to 0.5 revealed that 12 items had an ideal correct answer rate.

**Conclusion:**

We surmised that the low reliability of the mock examination was influenced by the timing of the test for the examinees and the inappropriate difficulty of the items. Our study suggested a methodology for the development of future case-based items for the Korean Nursing Licensing Examination.

## Introduction

The current Korean Nursing Licensing Examination consists of 295 items encompassing a total of 8 subjects. These items are generally based on existing baccalaureate curricula, on the learning objectives of major subjects for nursing, and on specifications outlined for the Korean Nursing Licensing Examination [1,2,3,4]. However, there is a need to transition from items that not only test rote learning skills and understanding of curricula to those that also test problem-solving skills using real-life clinical case studies and a nurse’s ability to interpret and apply the knowledge needed to perform the nursing role [5]. In past studies, researchers noted that major hurdles for new nurses, for which they were often unprepared, were integrative problem-solving skills, decision-making skills, and coping with situations requiring those skills [6,7]. Moreover, Suh et al. [8] reported that “items based on problem-solving skills that reflect the most up-to-date clinical situations” are needed and that “education teaching nurses how to cope with situations requiring integrative reasoning” should be implemented. Thus, this study aimed to develop case-based items testing the understanding and application of professional nursing knowledge and the problem-solving skills of nurses, and to validate them through item analysis, thereby suggesting that is possible to incorporate case-based items into the Korean Nursing Licensing Examination. The results of a mock examination using the newly developed case-based items are also summarized in this study.

## Methods

### Study design

A cross-sectional observational study was performed.

### Development process of items

***The nursing role:*** In a recent study, Park et al. [9] analyzed the nursing competencies required for the nursing role, and by doing so elucidated nurses’ role within healthcare. Their study outlined the job description of a nurse and listed the knowledge, techniques, and attitudes required for clinical performance. They found 8 duties, 49 tasks, and 303 job components that scored greater than 3 points on a 4-point scale. Herein, we used the same duties, tasks, and job components that were outlined in their study.

***Integrated nursing knowledge:*** Park et al. [10] conducted sub-categorization and linkage of the nursing job, comprising 8 duties and 49 task elements, and the learning outcomes of the Korean Nursing Licensing Examination courses from 7 baccalaureate nursing programs. Based on this study, a further sub-classification of the nursing job was performed by Kang et al. [5] in terms of task elements and learning outcomes, generating a total of 372 discrete elements of nursing knowledge required for the nursing job. Because both studies validated their results, we applied the 372 integrated elements of nursing knowledge as suggested by Kang et al. [5].

***Representative nursing cases:*** To obtain a representative sample of nursing case studies linking the components of the nursing job with integrated nursing knowledge and to develop case-based items, we first conducted 2 workshops for clinical nurses; the total number of participants was 23. Second, we collected clinical case studies from nurses, using a clinical case report form that we developed. Any personally identifiable information and hospital information were deleted, and factors such as gender, age, medical diagnosis, medical history, examination type, treatment plan, medications, and nursing intervention were included. Third, we asked for more details specific to the development of items for the Nursing Licensing Examination item. For instance, we included keywords essential to diseases and protocols for both standard medical examinations and examinations specific to the patient of a given case. For common drugs, the name, dosage, administration mode, and adverse drug reactions were collected. We also collected additional information concerning essential intervention skills and procedures for nursing (Table 1).

Fourth, among the collected case reports, our team and the clinical nurses who participated in the workshops selected a total of 40 case studies according to the completeness of information, the prevalence of the disease (favoring diseases that can be encountered in any hospital), and whether the learning goals, the nursing role, and the integrated knowledge were applicable to the general nursing job (Supplement 1).

***Development of case-based items:*** Fifth, through brainstorming of the representative nursing case studies by research fellows and clinical nurses, a total of 74 items were initially developed. Each item consisted of a 5-part structure: the heading, the clinical case, subordinate items, item branch, and multiple-choice answers. Each case was linked to 2-4 sub-items. All case-based items were developed following the criteria of the A-type best answer response and of the item development process [11,12].

The 74 items were linked to 8 nursing duties, to 30 nursing tasks, and to 54 elements of integrated nursing knowledge (Supplement 2).

Sixth, we tested the content validity of the 74 newly developed items in terms of the nursing role and the learning goals. The validation was performed by 60 examiners: 30 experts with more than 10 years of experience in various fields of nursing education and 30 preceptors with more than 3 years of clinical field experience who did not participate in the item development.

After several rounds of modification, deletion, and development based on the first-round assessment of content validity, the clinical nurses and our team developed a total of 50 items from 21 representative cases in accordance with the following selection criteria: having received a content validity index score of at least 3.0 for the nursing role and learning goals; being a prevalent case (reflecting conditions commonly encountered in the clinic); including knowledge required by new nurses; including current and evidence-based clinical roles of nurses; addressing appropriate learning goals; and being included in the specification of the current Korean Nursing Licensing Examination. A second-round assessment of content validity was performed for the refined set of items, and we validated and finalized the 50 items in terms of the nursing role and learning goals as appropriate (range, 3.0 to 3.9).

### Mock examination and item analysis

A mock examination consisting of the final 50 case-based items was implemented in 13 schools and taken by 741 students between October 26 and November 4, 2016. The students were given a total of 60 minutes for the examination, and each item was worth 2 points, giving a total of 100 possible points. Item analysis was conducted using 3 analytical methods. We first measured reliability, difficulty, and discrimination power, the latter 2 through classical test theory. We then measured reliability in terms of the Cronbach α, as well as the Spearman-Brown prediction method. To elucidate the factors that influenced reliability, we performed analysis of variance (ANOVA).

 Reliability index= {no. of items/(no. of items−1)}× {1−(score by item/total score)}

 Difficulty index= (no. of correct answers)/(no. of total answers) Item discrimination index= (upper 27% no. of correct answers– lower 27% no. of correct answers)/(0.27× no. of total answers)

 Item-total correlation index= no. of students× total no. of students (points for each question× score)–(total of points for each question× total score)/root {(no. of students× points)– sum of squares of points}× root {(no. of students× sum of squares of score)–square of total score}

We then used IRTPRO ver. 3.0 (Scientific Software International Inc., Skokie, IL, USA) to perform an analysis according to item response theory and assessed the following parameters: the item discrimination index, the item difficulty, the appropriateness index, and the item characteristic curve. We also interviewed a sample of students who took the mock examination to evaluate the response process validity.

### Ethical approval

This study received informed consent from all students and the approval of the institutional review board of the College of Nursing of Chungnam National University (2-1046881-A-N-01-201611-HR-050-02-03) after receiving informed consent from subjects.

## Results

### First-round content validity

For the first-round assessment of content validity, a score of 3.5 out of 4 points was reported (Supplement 3).

### Second-round content validity

The average range of content validity was 3.3 to 3.8 for the nursing role and 3.0 to 3.9 for the learning goals. Integrated nursing knowledge was linked to 34 items (Supplement 4).

### Final case-based items

The final case-based items were derived from the following nursing areas: 14 items related to 6 cases in adult nursing (breast cancer, lung cancer, thromboembolism, hepatic encephalopathy, cerebral hemorrhage, cervical cancer, and complex epilepsy); 7 items in basic nursing (dehydration and hydration with electrolyte imbalance, parotid gland cancer, and chronic obstructive pulmonary disease); 9 items in pediatric nursing (neonatal jaundice, acute gastroenteritis, respiratory disease, and nephrotic syndrome); 6 items in maternal nursing (placenta previa, abnormal uterine placental circulation, and preeclampsia); 7 items in mental health (schizophrenia and bipolar disease); and 7 items in community health (integrated case management of vulnerable families, public health centers, and industrial and workplace health).

### Item analysis

***Classical test theory:*** The responses of the students are shown in Supplement 5. The Cronbach alpha for the mock examination was relatively low (0.40). Using the Spearman-Brown prediction method, we predicted that at its current difficulty level, an examination with 450 items would show a reliability coefficient of 0.857. To delineate the factors that contributed to reliability, we performed ANOVA. The analysis showed that student-related factors contributed 0.98% towards overall reliability; item-related factors, 25.70%; and idiopathic factors, 73.31% (Table 2). According to classical test theory, the average item difficulty was 57.4% (12 items had an item difficulty of 80% to 100%; 13 items, 60% to 80%; and 25 items, below 60%). The mean item discrimination index was 0.19 (over 0.30 for 11 items and 0.20 to 0.29 for 15 items) (Supplement 6). The mean item-total correlation index was 0.18 (0.30 for 2 items; 0.20 to 0.29 for 22 items; and below 0.20 for 26 items). The results of the item content analysis, the job description, and an analysis of the 50 case-based items of the mock examination are summarized in Supplement 7.

**Fig. 1.**
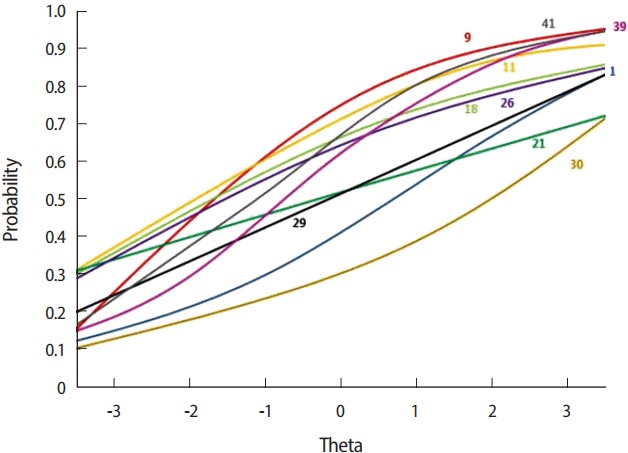
Item characteristic curve of moderate goodness of fit.

***Item response theory:*** The results of the metrics based on item response theory were as follows. The item discrimination parameter (in the logistic model) was none for 10 items (0.00), very low for 20 items (0.01 to 0.34), low for 12 items (0.35 to 0.64), moderate for 6 items (0.65 to 1.34), high for 1 item (1.35 to 1.69), and very high for 1 item (above 1.70). The item difficulty was very easy for 24 items (below −2.0), easy for 8 items (−2.0 to −0.5), medium for 6 items (−0.5 to 0.5), hard for 3 items (0.5 to 2.0), and very hard for 9 items (above 2.0) (Table 3). Based on the 2-parameter item response model, we used the 2.0 to 0.5 range for appropriateness testing and found that 12 items had an appropriate correct-answer rate (2, 3, 4, 5, 6, 12, 13, 23, 33, 37, 39, and 42). From the item characteristic curve, we found that 11 items (1, 9, 11, 18, 21, 26, 29, 30, 32, 39, and 41) had an average level of difficulty and appropriate discrimination power (Fig. 1).

***The response process validity of examinees:*** We measured the response process validity after students took the mock examination. We met 3 to 4 students from 3 groups and conducted a simple interview. We asked a mixture of open and closed questions, such as “How were the questions?” “How did the questions differ from those of the current Korean Nursing Licensing Examination?” “Were the case-based items answerable after some thought?” “Are the case studies included in the questions commonly encountered in real clinical settings?” and “Please tell us your opinion about the mock examination”.

An example of a typical response from the students is as follows: “The questions were useful in that they required us to integrate theory with practice. I felt that to get a higher mark on the test I would need to study a broader range of topics in greater depth, especially during practicums, as opposed to studying just the theoretical side. Although the Nursing Licensing Examination would become more difficult to study and to pass if the case-base items were to be included, I think it is the right step in development. The questions in the test required us to think critically. The case studies included in the questions were examples that we would commonly encounter during clinical practicums.”

## Discussion

In this study, we developed, assessed, and evaluated case-based items that tested problem-solving skills and clinical reasoning, in order to assess their potential use on the Korean Nursing Licensing Examination. In this study, we found that the relative contributions of each factor to the low reliability of the mock examination were as follows: student ability, 0.98%; item difficulty, 25.70%; and factors that could not be accounted for, 73.31%. The item difficulty of the mock examination according to classical test theory was 80% to 100% for only 24% of items, and 50% of the items had a high level of difficulty (less than 60%), indicating that students found the examination difficult. We suggest that other factors may have contributed to the low reliability. For instance, in most universities the mock examination was either held after the clinical practicum but before coursework modules began in the second semester or overlapped with mid-term exams if the clinical practicum was conducted in parallel to coursework modules. Some students had not completed their semester courses when they took the examination, meaning that there were differences in progress among exam-takers. Moreover, as the first takers of the examination, students were presumably unfamiliar with the case-based items, and the questions and the multiple-choice answers were not sufficiently refined, which resulted in an uneven difficulty level, poor discrimination power, and low item-total correlation, all of which may have contributed to low reliability. The assessment of the response process validity revealed that the exam-takers in general thought that the mock examination was difficult, as shown by the following 2 example examinee responses: “Some questions in the mock examination were difficult to answer because we hadn’t covered the relevant curricula yet” and “The mock examination was difficult because I hadn’t studied enough and because the questions required a lot of critical thinking.” The results of the metrics based on item response theory further support this, as 12 items had an item difficulty of hard or very hard. However, among the 50 items, students considered 24 items very easy and 8 items easy. The item discrimination parameter was none or almost none for 30 items and low for 12 items, revealing that there were many not-so-well-developed items. Although the overall reliability of the examination may increase with a greater number of items, a better option may be to refine the items so that they are at a more appropriate difficulty level and have a higher discrimination power, as opposed to increasing the number of the items, which could potentially reduce the quality of the examination. The appropriateness testing and the item characteristic curve showed that 12 and 11 items, respectively, had an appropriate correct-answer rate and discrimination power, as well as average difficulty. Since this study describes the development of the first-ever case-based items for the Korean Nursing Licensing Examination, we anticipate that there will be much improvement in the future in terms of the appropriateness, discrimination power, and difficulty of the items.

The significance of this technical report lies in suggesting the possibility of developing case-based items for the Korean Nursing Licensing Examination. Our findings also suggest that if nursing educational institutions adopt case-based problem-solving items in future examinations, examinees will be able to familiarize themselves with these item types relatively quickly and without much difficulty. We also anticipate that the clinical case forms for item development presented here will be productively utilized for the collection of new case studies from clinical nurses.

## Figures and Tables

**Table 1.** Clinical case input form (red color indicates added content)

Gender/age			Additional information for test item development	
Diagnosis	Key word		Knowledge nurses must have to care for the patient	
Chief complaint				
History (involved family history)				
Diagnostic test (Lab data, special tests, abnormal data)	Describe routine diagnostic tests and any addi- tional test that must be performed for this pa- tient		Intervention(s) that nurses must implement for the patient	
Treatment				
Drug	Name, dosage, route, side effects		Procedures that must be per formed for this patient	
Nursing problem				
Nursing intervention				

**Table 2.** Reliability and factors affecting reliability

(1) Reliability									
Students: differentiation 741.00 (no. of students)									
Items: random 50.00 (items)									
Pattern		Variance component		Levels		Signature		Rule	
Students		0.002		1.00		d		Tau only	
Items		0.001		50.00		r		Delta only	
Student-item		0.004		50.00		dr		Delta and delta	
Results									
S2(T) = 0.002									
S2(D) = 0.005									
S2(d) = 0.004									
Er2 = 0.402 (low-stakes test) – general reliability									
Phi = 0.332 (high-stakes test) – phi reliability of licensing exam									
(2) Spearman-Brown prediction									
No. of items	50	100	150	200	250	300	350	400	450
SD	11.87								
Reliability	0.4	0.571429	0.666667	0.727273	0.769231	0.8	0.823529	0.842105	0.857143
Standard error of measurement	9.194462464	7.770739	6.853148	6.198905	5.702164	5.308425	4.986397	4.71666	4.486438
(3) ANOVA table for run 1 (741 students, 50 items)									
Effect		Degree of freedom		Total	Sum of square		Mean square	Variance component	
Students		740		222.88599	222.88599		0.30120	0.00242 (0.98%)	
Items		49		2,303.79976	2,303.79976		47.01632	0.06321 (25.70%)	
Student-item		36,260		9,063.98599	6,537.30024		0.18029	0.18029 (73.31%)	
Mean		0.00000							
Total		37,049	9,063.98599						
Grand mean		0.00000							

SD, standard deviation; SE, standard error; ANOVA, analysis of variance.

**Table 3.** Item discrimination and item difficulty parameters according to item response theory (n=741)

No. of item	Item discrimination parameter		Item difficulty parameter	
1	0.55	Low	0.41	Medium
2	0.19	Very low	4.90	Very hard
3	0.13	Very low	1.56	Hard
4	0.32	Very low	-0.80	Easy
5	0.12	Very low	-13.97	Very easy
6	0.65	Moderate	-3.54	Very easy
7	0.52	Low	1.89	Hard
8	0.17	Very low	-3.43	Very easy
9	0.93	Moderate	-1.23	Easy
10	0.01	Very low	-23.77	Very easy
11	0.52	Low	-1.55	Easy
12	0.04	Very low	-44.88	Very easy
13	-0.46	None	-7.48	Very easy
14	0.27	Very low	-2.65	Very easy
15	-0.03	None	50.06	Very hard
16	0.31	Very low	-5.98	Very easy
17	1.43	High	-3.35	Very easy
18	0.40	Low	-0.95	Easy
19	0.57	Low	-4.39	Very easy
20	0.11	Very low	-0.12	Medium
21	0.27	Very low	-0.13	Medium
22	0.28	Very low	-7.44	Very easy
23	0.02	Very low	29.78	Very hard
24	-0.08	None	-0.56	Medium
25	-0.03	None	42.39	Very hard
26	0.44	Low	-0.85	Easy
27	0.38	Low	-3.98	Very easy
28	-0.13	None	8.61	Very hard
29	0.45	Low	0.08	Medium
30	0.50	Low	1.24	Hard
31	0.48	Low	2.49	Very hard
32	0.33	Low	0.26	Medium
33	0.22	Very low	4.83	Very hard
34	0.29	Very low	3.19	Very hard
35	1.94	Very high	-3.01	Very easy
36	0.22	Very low	3.89	Very hard
37	-0.07	None	-9.59	Very easy
38	0.70	Moderate	-2.30	Very easy
39	0.76	Moderate	-0.79	Easy
40	0.77	Moderate	-2.84	Very easy
41	0.71	Moderate	-0.77	Easy
42	0.56	Low	-1.98	Easy
43	0.19	Very low	-3.96	Very easy
44	0.24	Very low	-3.23	Very easy
45	-0.18	None	-6.23	Very easy
46	0.20	Very low	-4.27	Very easy
47	0.26	Very low	-3.93	Very easy
48	-0.12	None	-24.54	Very easy
49	-0.23	None	-9.76	Very easy
50	-0.17	None	-5.99	Very easy

## Supplementary materials

Supplement 1. Example of a representative nursing case.

Supplement 2. Example of the first-round case form for item development.

Supplement 3. Example of the first-round assessment of content validity.

Supplement 4. Example of the second-round assessment of content validity.

Supplement 5. Data file is available from https://doi.org/10.7910/DVN/ECDLXG.

Supplement 6. Difficulty and discrimination by classical test theory.

Supplement 7. Item content, job description, and analysis of 50 case-based items for the mock examination.

Supplement 8. Audio recording of the abstract.

## References

[1] Lee HY, Kim CJ, Lee SJ, Park HR, Lee IS, Kim HJ, Park YM (2005). A study of the validity of the Korean Nurses’ Licensing Examination. J Educ Eval Health Prof.

[2] Kim KS, Kang YH, Koo HY, Kwan MJ, Kim NC, Kim OS, Park JS, Shin HS, Ahn OH, Jang SO (2013). Systematical improvement on the subjects of Korea Nursing License Examination.

[3] Park HR, Kim KH, Kang YS, Kim SY, Park EH (2011). Comparative study on the educational contents of Korea Nursing License Examination by school system.

[4] Korea Health Personnel Licensing Examination Institute (2014). Specific area for developing test item pool of Korean Nursing Licensing Examination.

[5] Kang SY, Kim KS, Kim GH, Park IS, Park HS, Suh YO, Ahn SY Development of nursing knowledge statement based on job description for new graduate nurses in Korea.

[6] Park IS, Park IH, Kim SJ, Suh YO, Park HS, Park EH (2010). Job analysis of the new nurses for national licensing examination.

[7] Lim NY, Song JH (2007). Delphi study on introduction of practical skills test in national examination for nursing licensure. J Korean Acad Fundam Nurs.

[8] Suh YO, Kang SY, Park IS, Shin SJ, Hwang SY (2016). Status of the Korean nursing licensure examination and suggestion for improvement.

[9] Park IS, Suh YO, Park HS, Ahn SY, Kang SY, Ko IS (2016). The job analysis of Korean nurses as a strategy to improve the Korean Nursing Licensing Examination. J Educ Eval Health Prof.

[10] Park IS, Suh YO, Park HS, Ahn SY, Kang SY, Kim KS (2016). Relevance of the test content of the Korean Nursing Licensing Examination to nursing job. J Educ Eval Health Prof.

[11] National Board of Medical Examiners (2002). Constructing written test questions for the basic and clinical sciences.

[12] Harding MM, Snyder JS, Preusser BA (2016). Winningham’s critical thinking cases in nursing.

