# Supplementary material for: Item development process and analysis of 50 case-based items for implementation on the Korean Nursing Licensing Examination
Source: J Educ Eval Health Prof. 2017 Sep 11;14:20. doi: 10.3352/jeehp.2017.14.20 (PMC5729210; doi:10.3352/jeehp.2017.14.20)
Supplement: Supplementary file 1 — Supplement 1. Example of a representative nursing case. [file jeehp-14-20-suppl1.pdf]

Supplement 1. Example of a representative nursing case

Gender/age	2) Female	45 years old
Diagnosis	Breast ca. Lt. (Female hormone (estrogen), lymphedema, heredity)	
Major symptoms	Breast mass that appeared without pain	
Medical history (including past history and family history)	* Past history (PH)<br>Blood pressure/diabetes/hepatitis/tuberculosis(-/-/-/-)<br>GB polyp, s/p Lap. cholecystectomy<br>* Family history (FH): None	
Diagnostic tests (clinical pathology examination, special tests), abnormal data	* General examination<br>1. Complete blood cell count (CBC), blood chemistry examination, blood coagulation test<br>2. ECG, Chest X-ray<br>3. Imaging test relating to breast (mammography, breast ultrasound, breast MRI)<br>: A 2.0 × 1.7 cm enhancing mass in L 12 hr direction.<br>- distance from nipple: 2.0 cm<br>; C6, biopsy-proven malignancy<br>4. PET/CT (checking if a remote metastasis exists)<br>: Hypermetabolic known malignant tumor in mid-upper portion of left breast.<br>No definite evidence of LN or distant metastasis.<br>5. Histopathologic examination<br>(Immunohistochemical staining: estrogen receptor, progesterone receptor, HER-2 presence/absence)<br>: Breast, 12 o'clock direction, left, needle biopsy;<br>Invasive ductal carcinoma, moderately differentiated.<br>[Immunohistochemistry]<br>Estrogen receptor (+), progesterone receptor (+), C-erbB2 (3+)<br>* Patient aged 60 or older: pulmonary function test, echocardiography<br>* When remote metastasis is suspected:<br>- Consider CT or MRI according to the suspected areas<br>ex) Chest and abdomen CT, and bone metastasis site MRI, etc.	
Major treatment methods	* Surgery<br>Breast conservation therapy and sentinel lymph node (breast wide excision and sentinel node biopsy, Lt.)<br>- Pathologic outcome<br>: Invasive ductal carcinoma stage IA, pT1N0M0, LN 0/5)<br>: ER(+), PR(+), C-erbB2(3 +)<br>* Adjuvant radiation therapy<br>Post op RTx on the Lt. breast → 5040 cGy/28 times/5.5 weeks.<br>* Adjuvant chemotherapy<br>Adriamycin/cyclophosphamide × 4 times (3-week intervals)<br>* Targeted therapy (if HER-2-positive)<br>Trastuzumab × 1 year (3-week intervals)<br>* Anti-hormone therapy (if ER- or PR-positive)<br>Tamoxifen × 5 years (30 minutes after each meal every morning, per oral)	

Major therapeutic drugs (Name of drugs and adverse reactions that nurses should know)	* Anti-cancer drugs<br>Adriamycin 60 mg/BSA, IV<br>Cyclophosphamide 600 mg/BSA, IV<br>Side effect: depressed marrow (neutropenia, anemia, thrombocytopenia), nausea/vomiting, stomatitis, hair loss, diarrhea/constipation, cardiac dysfunction, liver failure, fatigue/helplessness, and possibility of menopause<br>* Targeted therapy (stopping signaling related to cancer cell growth caused by HER-2 receptor overexpression)<br>Trastuzumab (Herceptin) initially 8 mg/kg loading administration and → maintaining 6 mg/kg<br>Adverse reactions: hypersensitivity reaction (hives, urtication, giddiness, dyspnea, chill, and pyrexia), cardiac dysfunction (need to check cardiac function through regular echocardiography!)<br>*Anti-hormone therapy (therapy to stop the influence of female hormones on breast cancer)<br>Tamoxifen 20 mg (1T), per oral<br>- Working principle: Inhibiting cell growth by combining with estrogen receptor instead of estrogen in breast cancer → reduction of local recurrence and occurrence of remote metastasis & reduction of cancer in opposite breast<br>- Adverse reactions: flush, heat sensation, decreased sexual desire, skin dryness, vaginal dryness, dyspareunia, colporrhagia and vaginal discharge, endometrial hyperplasia (gynecological examination is recommended at intervals of 6 months to one year), venous and arterial thrombosis, and retinopathies
Major nursing problems	Adverse reaction of chemotherapy (neutropenia, infection)<br>Lymphedema caused by axillary lymph node resection<br>Cardiotoxicity caused by chemotherapy and targeted therapy<br>Adverse reaction of taking tamoxifen<br>Sense of loss or depression caused by mastectomy
Nursing interventions	Surgery education (Precautions according to surgical methods, Daily life management, arm exercising method, lymphedema management)<br>Anti-cancer education (Management of adverse reaction during chemotherapy)<br>Education on taking anti-hormone drugs (Management of adverse reaction)
What a nurse must know to care for this patient	Knowledge about treatment of breast cancer<br>Surgery, anti-cancer, radiation, targeted therapy, and anti-hormone drugs, etc.
Behavior and interventions that the nurse must perform for this patient	Checking family history (FH)<br>Education on the management of adverse reaction to each treatment

(Please write down any procedure that must be observed during the nursing care of this patient.)	Arm exercising method and lymphedema management after mastectomy<br>Self-nursing during the period of neutrophil reduction after chemotherapy (Infection control method)
