# Supplementary material for: Item development process and analysis of 50 case-based items for implementation on the Korean Nursing Licensing Examination
Source: J Educ Eval Health Prof. 2017 Sep 11;14:20. doi: 10.3352/jeehp.2017.14.20 (PMC5729210; doi:10.3352/jeehp.2017.14.20)
Supplement: Supplementary file 2 — Supplement 2. Example of the first-round case form for item development. [file jeehp-14-20-suppl2.pdf]

Supplement 2. Example of the first-round case form for item development

<table>
<tr><td colspan="4">Representative Nursing Case 1-1 (Examination Question 1-2 link)</td></tr>
<tr><td rowspan="2">Job</td><td rowspan="2">F3
(Transfusion nursing)</td><td>Area of learning objectives (category)</td><td>3. Cancer management</td></tr>
<tr><td>Area (division)</td><td>2) Anticancer therapy nursing</td></tr>
<tr><td>Major area</td><td>Basic, adult nursing area</td><td>Nutrition /metabolism/excretion Nursing</td><td>Breast cancer</td></tr>
<tr><td>Integrated nursing knowledge</td><td colspan="3">Execution of appropriate tests and procedure for transfusion</td></tr>
</table>

A 45-year-old breast cancer patient visited an emergency room with stomatitis and fever as chief complaints on the seventh day after the administration of anti-cancer drugs started. The result of a CBC test was Hb 9 g/dL, Hct 18%, and platelets 5000/mm^3. Therefore, 2 units of packed red blood cells were prescribed.

Which are the appropriate general examinations that should be performed to confirm compatibility before blood transfusion?

1) Cross-matching test, HBsAg Test
2) Antibody screening test, HIV test
3) ABO and Rh type, cross-matching test
4) ABO and Rh type, genetic testing
5) Antibody screening test, cold agglutinin test

Knowledge level of question	Memorizing type	Interpreting type	Problem-solving type	Answer	Level of difficulty
Knowledge level of question	√			3	Low

Basis	·Blood compatibility test before transfusion<br>·It should be not confused with the pretest for a blood donor.<br>·Knowledge of transfusion procedures and blood test results, nursing according to transfusion guide
Reference	Kim et al., 2013, Korean Journal of Adult Nursing, 1062-1063, Sumunsa
