# Supplementary material for: Item development process and analysis of 50 case-based items for implementation on the Korean Nursing Licensing Examination
Source: J Educ Eval Health Prof. 2017 Sep 11;14:20. doi: 10.3352/jeehp.2017.14.20 (PMC5729210; doi:10.3352/jeehp.2017.14.20)
Supplement: Supplementary file 3 — Supplement 3. Example of the first-round assessment of content validity. [file jeehp-14-20-suppl3.pdf]

Supplement 3. Example of the first-round assessment of content validity

Case 1

1. A 45-year-old breast cancer patient visited an emergency room with stomatitis and fever as chief complaints on the seventh day after the administration of anti-cancer drugs started. The result of a CBC test was Hb 9 g/dL, Hct 18%, and platelets 5000/mm_3. Therefore, 2 units of packed red blood cells were prescribed.
2. Question before modification:

Which are the appropriate general examinations that should be performed to confirm compatibility before blood transfusion?

1) Cross-matching test, HBsAg Test
2) Antibody screening test, HIV test
3) ABO and Rh type, cross-matching test
4) ABO and Rh type, genetic testing
5) Antibody screening test, cold agglutinin test

3. Job of corresponding nurse: F3 Transfusion Nursing
4. Corresponding learning objective: 3. Cancer Management
5. Mean job validity: 3.9
6. Mean learning objective validity: 3.2
7. Advice of expert: Learning objective 5-2) seems more appropriate.
8. Modification of case
9. Final question: Question modification

Case 2

1. A 45-year-old breast cancer patient visited an emergency room with stomatitis and fever as chief complaints on the seventh day after the administration of anti-cancer drugs started. The result of a CBC test was Hb 9 g/dL, Hct 18%, and platelets 5000/mm_3. Therefore, 2 units of packed red blood cells were

prescribed.

2. Question after modification:

Which Hb level would be normally expected after the transfusion?
1) 9 g/dL
2) 10 g/dL
3) 11 g/dL
4) 12 g/dL
5) 13 g/dL

3. Job of corresponding nurse: F3 Transfusion Nursing

4. Corresponding learning objective: 2. Activity-Rest Nursing

5. Mean job validity: 3.9

6. Mean learning objective validity: 3.1

7. Advice of expert: Learning objective 5-2) seems more appropriate.

8. Modification of case

9. Final question: question modification
