# Supplementary material for: Item development process and analysis of 50 case-based items for implementation on the Korean Nursing Licensing Examination
Source: J Educ Eval Health Prof. 2017 Sep 11;14:20. doi: 10.3352/jeehp.2017.14.20 (PMC5729210; doi:10.3352/jeehp.2017.14.20)
Supplement: Supplementary file 4 — Supplement 4. Example of the second-round assessment of content validity. [file jeehp-14-20-suppl4.pdf]

Supplement 4. Example of the second-round assessment of content validity

Case 1

An invasive ductal carcinoma (IDC) was found in a 45-year-old female, and a breast conservation procedure, lymphoidectomy, and anticancer therapy will be performed.

Question after modification: Which of the following is an appropriate nursing intervention to prevent lymphedema after mastectomy?

1) Doing a shoulder rotation exercise

2) Lifting the arm on the side of the operated breast

3) Evaluating sensation at the surgical site

4) Doing arm and finger movements involving flexion and extension

5) Contracting the abdominal muscles by taking a deep breath

Job: C4. Preoperative/intraoperative/postoperative nursing

Corresponding learning objective: 4. Body fluid imbalance / dysuria

Mean job validity: 3.5

Mean learning objective validity: 2.8

Advice of expert:

Final question: Question selection

Case 2

An invasive ductal carcinoma (IDC) was found in a 45-year-old female, and a breast conservation procedure, lymphoidectomy, and anticancer therapy will be performed. Since the patient's tumor is estrogen and progesterone receptor-positive, an estrogen receptor blocker (tamoxifen) will be injected.

Question after modification: Which behavior should be warned against when educating the patient about the risk of adverse reactions?

1) High-protein diet

2) Weight gain

3) Smoking

4) Drinking

5) Flexibility exercises

Job: F1. Administering drugs

Corresponding learning objective: 6. Cardiovascular/blood disorders

Mean job validity: 3

Mean learning objective validity: 3

Advice of expert:

Final question: Question selection
