# Supplementary material for: Item development process and analysis of 50 case-based items for implementation on the Korean Nursing Licensing Examination
Source: J Educ Eval Health Prof. 2017 Sep 11;14:20. doi: 10.3352/jeehp.2017.14.20 (PMC5729210; doi:10.3352/jeehp.2017.14.20)
Supplement: Supplementary file 5 — Supplement 6. Difficulty and discrimination by classical test theory. [file jeehp-14-20-suppl6.pdf]

**Supplement 6.** Difficulty and discrimination by classical test theory (item=50)

| Difficulty (%) | No. of item (%) | Discrimination | No. of item (%) |
|---|---|---|---|
| 90–100 | 4 (8.0) | Above 0.30 | 11 (22.0) |
| 80–89 | 8 (16.0) | 0.20–0.29 | 15 (30.0) |
| 70–79 | 6 (12.0) | 0.10–0.19 | 15 (30.0) |
| 60–69 | 7 (14.0) | Under 0.10 | 9 (18.0) |
| 50–59 | 6 (12.0) | - | - |
| 40–49 | 5 (10.0) | - | - |
| 30–39 | 4 (8.0) | - | - |
| 20–29 | 7 (14.0) | - | - |
| 10–19 | 1 (2.0) | - | - |
| 0–9 | 2 (4.0) | - | - |
