# Supplementary material for: Item development process and analysis of 50 case-based items for implementation on the Korean Nursing Licensing Examination
Source: J Educ Eval Health Prof. 2017 Sep 11;14:20. doi: 10.3352/jeehp.2017.14.20 (PMC5729210; doi:10.3352/jeehp.2017.14.20)
Supplement: Supplementary file 6 — Supplement 7. Item content, job description, and analysis of 50 case-based items for the mock examination. [file jeehp-14-20-suppl7.pdf]

Supplement 7. Item content, job description, and analysis of 50 case-based items for the mock examination

Case 1: Questions 1–2

An invasive ductal carcinoma (IDC) was found in a 45-year-old woman, and a breast conservation procedure, lymphoidectomy, and anticancer therapy will be performed.

Job	C4. Preoperative/intraoperative/postoperative nursing	Area of learning objectives (category / division)	Body fluid imbalance / dysuria
Integrated nursing knowledge	Observation of the incidence of postoperative complications and complications in preventive nursing		

1. Which of the following is an appropriate nursing intervention to prevent lymphedema after mastectomy?

1) Doing a shoulder rotation exercise

2) Lifting the arm on the side of the operated breast

3) Evaluating sensation at the surgical site

4) Doing arm and finger movements involving flexion and extension

5) Contracting the abdominal muscles by taking a deep breath

Category	High (27%)	Middle (46%)	Low (27%)	Total	Correct answer rate	Discrimination index	Item-total correlation
No. of correct answers	121	154	57	332	45%	0.32	0.26
No. of wrong answers	79	187	143	409			
Total	200	341	200	741			

Response distribution	No. 1	No. 2	No. 3	No. 4	No. 5	Unanswered	Duplicated	Total
Upper 27%	36	121	18	12	13	0	0	200
Lower 27%	53	57	54	23	13	0	0	200
Total	89	178	72	35	26	0	0	400

`	F1. Administering drugs	Area of learning objectives (category / division)	Cardiovascular/ blood disorders
Integrated nursing knowledge	Administration of medicine according to a systems-based approach to disease and medication-related nursing		

2. Since the patient's tumor is estrogen and progesterone receptor-positive, an estrogen receptor blocker (tamoxifen) will be injected. Which behavior poses a high risk of an adverse reaction?

1) High-protein diet 2) Weight loss 3) Smoking 4) Drinking 5) Flexibility exercises

Category	High (27%)	Middle (46%)	Low (27%)	Total	Correct answer rate	Discrimination index	Item-total correlation
No. of correct answers	80	93	40	213	29%	0.20	0.16
No. of wrong answers	120	248	160	528			
Total	200	341	200	741			

Response Distribution	No. 1	No. 2	No. 3	No. 4	No. 5	Unanswered	Duplicated	Total
Upper 27%	45	42	80	28	5	0	0	200

Lower 27%	51	73	40	28	8	0	0	200
Total	96	115	120	56	13	0	0	400

Case 2: Questions 3–5

Cold medicine was prescribed to a 65-year-old male patient for 3 months for a mucous cough. Since his symptoms did not improve, a chest X-ray and computed tomography scan were taken, and a mass of 3 cm or larger was confirmed in the right upper lobe. After a bronchoscopic evaluation, he was diagnosed with squamous cell carcinoma. The peribronchial lymph node was invaded and there was no distant metastasis.

Job	F4. Managing patients who receive anticancer therapy	Area of learning objectives (category / division)	Respiratory failure
Integrated nursing knowledge	Nursing for patients with neoplastic disease by system		

3. What stage of lung cancer is this patient classified as having?

1) $T_0N_1M_0$ 2) $T_1N_1M_0$ 3) $T_2N_1M_0$ 4) $T_3N_2M_1$ 5) $T_3N_2M_0$

Category	High (27%)	Middle (46%)	Low (27%)	Total	Correct answer rate	Discrimination index	Item- total correlation
No. of correct answers	99	161	73	333	45%	0.13	0.13
No. of wrong answers	101	180	127	408			
Total	200	341	200	741			

Response distribution	No. 1	No. 2	No. 3	No. 4	No. 5	Unanswered	Duplicated	Total
Upper 27%	5	57	99	10	29	0	0	200
Lower 27%	5	70	73	12	40	0	0	200
Total	10	127	172	22	69	0	0	400

Job	E6. Managing drainage tubes	Area of learning objectives (category / division)	Respiratory failure
Integrated nursing knowledge	Management of drainage tubes at surgical sites		

4. This patient underwent a thoracoscopic right upper lobectomy and mediastinal lymph node dissection. After the operation, a thoracic tube was inserted and a water-sealed thoracic drainage device was placed. If tidaling in the water-sealed chamber is absent, what is the first thing the nurse should do?

1) Lock the thoracic tube

2) Exchange the sealed bottle

3) Change the posture of the patient

4) Leave it as it is, because it is only a temporary situation

5) Observe whether there are bubbles in the sealed bottle

Category	High (27%)	Middle (46%)	Low (27%)	Total	Correct answer rate	Discrimination index	Item-total correlation
No. of correct answers	143	187	87	417	56%	0.28	0.20
No. of wrong answers	57	154	113	324			

Total	200	341	200	741			

Response distribution	No. 1	No. 2	No. 3	No. 4	No. 5	Unanswered	Duplicated	Total
Upper 27%	22	1	143	2	32	0	0	200
Lower 27%	43	4	87	1	65	0	0	200
Total	65	5	230	3	97	0	0	400

Job	F4. Managing patients who receive anticancer therapy	Area of learning objectives (category / division)	Comfort alteration
Integrated nursing knowledge	Nursing cancer patients according to the treatment method		

5. This patient underwent a chemoport insertion procedure, and then paclitaxel-carboplatin were administered 4 times as adjuvant chemotherapy. During chemotherapy, extravasation of about 1 mL occurred and the patient complained of pain. What would be the highest-priority nursing intervention in this situation?

1) Injecting an antidote

2) Taking a picture of the affected site

3) Stopping the drug injection

4) Placing a compressive sterilizing dressing

5) Placing a cold pack or hot pack

Category	High (27%)	Middle (46%)	Low (27%)	Total	Correct answer rate	Discrimination index	Item-total correlation
No. of correct answers	182	286	154	622	84%	0.14	0.15
No. of wrong answers	18	55	46	119			

Total	200	341	200	741			

Response distribution	No. 1	No. 2	No. 3	No. 4	No. 5	Unanswered	Duplicated	Total
Upper 27%	3	2	182	4	9	0	0	200
Lower 27%	12	8	154	10	16	0	0	200
Total	15	10	336	14	25	0	0	400

Case 3: Questions 6-7

A 77-year-old female patient is taking enoxaparin sodium (Clexane), which is an anticoagulant, due to small bowel obstruction caused by thromboembolism of the arteria mesenterica superior, and received L-tube irrigation because she showed bloody stool. Her hemoglobin level was 5.9 mg/dL, and her pulse increased and breathing became erratic while she complained that she felt tired.

Job	C1. Monitoring the patient's status	Area of learning objectives (category / division)	Cardiovascular disorders / hematologic disorders
Integrated nursing knowledge	Nursing diagnosis and determining the order of priority based on collected data		

6. What should be the highest-priority assessment based on her symptoms and medical history?

1) Blood pressure 2) Degree of pain 3) Na^+ level 4) Body temperature 5) Fall risk score

Category	High (27%)	Middle (46%)	Low (27%)	Total	Correct answer rate	Discrimination index	Item-total correlation
Upper 27%	194	308	162	664	90%	0.16	0.16
Lower 27%	6	33	38	77			
Total	200	341	200	741			

Response	No. 1	No. 2	No. 3	No. 4	No. 5	Unanswered	Duplicated	Total

distribution								
Upper 27%	194	2	3	0	1	0	0	200
Lower 27%	162	5	19	13	1	0	0	200
Total	356	7	22	13	2	0	0	400

Job	E2. Maintaining circulation	Area of learning objectives (category / division)	Cardiovascular disorders / hematologic disorders
Integrated nursing knowledge	Nursing for patients with cardiovascular disorders		

7. The CVP of this patient was measured to be 10 cm H_2O. What should be done first for this patient?

1) Prepare the patient for a blood transfusion

2) Have the patient assume a semirecumbent position

3) Check whether the patient has bloody stool

4) Administer 5% dextrose fluid

5) Measure bleeding time and blood coagulation time

Category	High (27%)	Middle (46%)	Low (27%)	Total	Correct answer rate	Discrimination index	Item-total correlation
No. of correct answers	82	88	40	210	28%	0.21	0.20
No. of wrong answers	118	253	160	531			
Total	200	341	200	741			

Response distribution	No. 1	No. 2	No. 3	No. 4	No. 5	Unanswered	Duplicated	Total
Upper 27%	82	55	10	16	37	0	0	200
Lower 27%	40	74	9	37	40	0	0	200
Total	122	129	19	53	77	0	0	400

Case 4: Questions 8–10

A 69-year-old man had a transarterial chemoembolization (TACE) 2 years ago due to liver cancer and also suffers from esophageal varices (EV). Starting on the evening of the day he was admitted to the hospital, his consciousness seriously deteriorated to the degree that he did not respond when called. Lab reports showed the following results: hemoglobin, 11.6; CRP, 0.49; BUN/Cr, 12.8/0.84; albumin, 2.7, Na/K, 140/4.4; AST/ALT, 55/30; ammonia, 357 μg/dL, and INR, 1.47.

<table>
<tr><td>Job</td><td>F1. Administering drugs</td><td rowspan="2">Area of learning objectives (category / division)</td><td rowspan="2">Intake / absorption / metabolic disorders</td></tr>
<tr><td>Integrated nursing knowledge</td><td>Administration of medicine according to a systems-based approach to disease and medication-related nursing</td></tr>
</table>

8. A doctor prescribed 30 mL of lactulose (Cephulac). What is the purpose of administering this drug?

1) Nausea

2) Vomiting

3) Increasing the amount of urine

4) Reducing the ammonia level

5) Relieving constipation

Category	High (27%)	Middle (46%)	Low (27%)	Total	Correct answer rate	Discrimination index	Item-total correlation
No. of correct answers	146	220	106	472	64%	0.20	0.18
No. of wrong answers	54	121	94	269			
Total	200	341	200	741			

Response distribution	No. 1	No. 2	No. 3	No. 4	No. 5	Unanswered	Duplicated	Total
Upper 27%	0	2	6	146	46	0	0	200
Lower 27%	10	12	19	106	53	0	0	200
Total	10	14	25	252	99	0	0	400

Job	D2. Nutrition management	Area of learning objectives (category / division)	Intake / absorption / metabolic disorders
Integrated nursing knowledge	Assessment and management of nutrition issues by disease		

9. The intake of which nutrient should be adjusted to reduce the ammonia level of this patient?

1) Carbohydrate 2) Fat 3) Protein 4) Salt 5) Calcium

Category	High (27%)	Middle (46%)	Low (27%)	Total	Correct answer rate	Discrimination index	Item-total correlation
No. of correct answers	172	254	112	538	73%	0.30	0.30
No. of wrong answers	28	87	88	203			
Total	200	341	200	741			

Response distribution	No. 1	No. 2	No. 3	No. 4	No. 5	Unanswered	Duplicated	Total
Upper 27%	2	11	172	12	3	0	0	200
Lower 27%	10	19	112	46	13	0	0	200
Total	12	30	284	58	16	0	0	400

Job	C2. Emergency response	Area of learning objectives (category / division)	Immunologic / physical injuries
Integrated nursing knowledge	Emergency nursing according to the situation		

10. Thirty minutes after a Sengstaken-Blakemore tube was inserted due to esophageal variceal bleeding, the nurse found that the patient’s breathing was becoming unstable. What is the first thing the nurse should do?

1) Remove the tube

2) Report this observation to the doctor

3) Check the patient’s oxygen saturation

4) Remove the pressure of the tube on the esophagus

5) Check whether the tube blocks the airway

Category	High (27%)	Middle (46%)	Low (27%)	Total	Correct answer rate	Discrimination index	Item- total correlation
No. of correct answers	125	181	101	407	55%	0.12	0.11
No. of wrong answers	75	160	99	334			
Total	200	341	200	741			

Response distribution	No. 1	No. 2	No. 3	No. 4	No. 5	Unanswered	Duplicated	Total
Upper 27%	11	1	52	11	125	0	0	200
Lower 27%	6	5	80	8	101	0	0	200
Total	17	6	132	19	226	0	0	400

Case 5: Questions 11–12

A 52-year-old man was taken to the emergency room while he was painting. Before he started painting, it is said that he complained of a headache. Suspecting a rupture of the posterior cerebellar artery, an embolization was performed. A subarachnoidal hemorrhage was confirmed, so an external ventricular drainage (EVD) tube was placed. The patient shows a Na level of 130 mEq/L and 3% physiological saline is administered into the central vein. In addition, mannitol and an antiemetic drug are being administered.

Job	E4. Nursing regarding the maintenance of the senses	Area of learning objectives (category / division)	Cognitive / neurological disorders
Integrated nursing knowledge	Nursing of patients with increased intracranial pressure (IICP)		

11. Which nursing intervention would prevent increased intracranial pressure (IICP)?

1) Injecting dextrose fluid

2) Maintaining the body temperature at 37.5℃

3) Maintaining a supine position

4) Administrating a laxative preventively

5) Maintaining blood pressure below 120/80 mm Hg

Category	High (27%)	Middle (46%)	Low (27%)	Total	Correct answer rate	Discrimination index	Item-total correlation
No. of correct answers	161	234	109	504	68%	0.26	0.25
No. of wrong answers	39	107	91	237			
Total	200	341	200	741			

Response distribution	No. 1	No. 2	No. 3	No. 4	No. 5	Unanswered	Duplicated	Total
Upper 27%	3	3	6	161	27	0	0	200
Lower 27%	8	3	22	109	58	0	0	200
Total	11	6	28	270	85	0	0	400

Job	E4. Nursing regarding the maintenance of the senses	Area of learning objectives (category / division)	Cognitive / neurological disorders
Integrated nursing knowledge	Nursing of patients with increased intracranial pressure (IICP)		

12. Which vital sign indicates increased intracranial pressure (IICP) in this patient?

1) Blood pressure of 120/80 mm Hg

2) Breath rate of 20 breaths per minute

3) Pulse of 72 beats per minute

4) Body temperature of 37℃

5) Pulse pressure of 60 mm Hg

Category	High (27%)	Middle (46%)	Low (27%)	Total	Correct answer rate	Discrimination index	Item-total correlation
No. of correct answers	184	293	165	642	87%	0.10	0.13
No. of wrong answers	16	48	35	99			
Total	200	341	200	741			

Response distribution	No. 1	No. 2	No. 3	No. 4	No. 5	Unanswered	Duplicated	Total
Upper 27%	2	2	2	10	184	0	0	200
Lower 27%	5	4	9	17	165	0	0	200
Total	7	6	11	27	349	0	0	400

Case 6: Questions 13–14

A 57-year-old woman who underwent hysterectomy due to endometrial cancer recently had a peritonectomy. She was admitted to the hospital since she felt listless throughout the body and mentally drowsy for the last few days. On the next day of admission, her head and eyes turned upward and she experienced a general seizure and eyeball deviation, twisting her body for about 1 minute, and then fell into a stupor for 20 minutes. She then showed the second symptom of epilepsy for about 1 minute. During the seizure, her vital signs were as follows: 190/100 mm Hg; 130 beats per minute; 25 breaths per minute; 36.9°C; SpO_2, 96%; Na, 154; K, 2.7; albumin, 2.6; Ca, 6.8; and Mg, 1.1.

Job	C1. Monitoring the patient's status	Area of learning objectives (category / division)	Cognitive / neurological disorders
Integrated nursing knowledge	Administration of medicine according to a systems-based approach to disease and medication-related nursing		

13. Which lab test parameter should be monitored during the administration of phenytoin as an antiepileptic agent?

1) Na 2) K 3) Hematocrit 4) Albumin 5) Hemoglobin

Category	High (27%)	Middle (46%)	Low (27%)	Total	Correct answer rate	Discrimination index	Item-total correlation
No. of correct answers	3	14	9	26	4%	-0.02	-0.04
No. of wrong answers	197	327	191	715			
Total	200	341	200	741			

Response distribution	No. 1	No. 2	No. 3	No. 4	No. 5	Unanswered	Duplicated	Total
Upper 27%	32	149	3	13	3	0	0	200
Lower 27%	49	113	10	18	9	1	0	200
Total	81	262	13	31	12	1	0	400

Job	B3. Prevention and management of injuries and accidents	Area of learning objectives (category / division)	Cognitive / neurological disorders
Integrated nursing knowledge	Response to patient safety problems		

14. What is the first thing the nurse should do during the patient's seizure?

1) Maintain the patient in a supine position

2) Remove dangerous items on the bed

3) Use a restraining band to prevent a fall

4) Perform an intubation to establish a free airway

5) Place a tongue depressor to prevent curling of the tongue

Category	High (27%)	Middle (46%)	Low (27%)	Total	Correct answer rate	Discrimination index	Item-total correlation
No. of correct answers	165	229	103	497	67%	0.31	0.25
No. of wrong answers	35	112	97	244			
Total	200	341	200	741			

Response distribution	No. 1	No. 2	No. 3	No. 4	No. 5	Unanswered	Duplicated	Total
Upper 27%	1	165	0	5	29	0	0	200
Lower 27%	8	103	3	14	71	0	1	200
Total	9	268	3	19	100	0	1	400

Case 7: Questions 15–16

A 24-year-old man suddenly lost consciousness and collapsed on the street while hiking across the country with his friends during summer vacation, and he was taken to the emergency room of a hospital by ambulance. When he came to the hospital, he kept trying to sleep, spoke gibberish, and showed symptoms of vomiting from time to time. His lips and tongue looked cracked and dry. His body temperature was 40.7°C and pulse and breathing were 116 beats/min and 16 breaths/min, respectively. His blood pressure was measured to be 90/60 mm Hg.

Job	D1. Provision of individual hygiene	Area of learning	Safety

Integrated nursing knowledge	Nursing interventions for maintenance of water-electrolyte balance	objectives (category / division)	requirements

15. Which nursing intervention should be performed with the highest priority by the nurse?

1) Administering antibiotics

2) Maintaining an environment with high humidity

3) Measuring vital signs every 4 hours

4) Replenishing water and electrolytes by administering intravenous fluids

5) When bathing, starting to clean from the area close to the heart in tepid water

Category	High (27%)	Middle (46%)	Low (27%)	Total	Correct answer rate	Discrimination index	Item-total correlation
No. of correct answers	167	295	157	619	84%	0.05	0.06
No. of wrong answers	33	46	43	122			
Total	200	341	200	741			

Response distribution	No. 1	No. 2	No. 3	No. 4	No. 5	Unanswered	Duplicated	Total
Upper 27%	0	0	8	167	25	0	0	200
Lower 27%	6	4	10	157	22	1	0	200
Total	6	4	18	324	47	1	0	400

Job	D1. Provision of individual hygiene	Area of learning objectives (category / division)	Safety requirements
Integrated nursing knowledge	Individual hygiene nursing		

16. Which procedure is correct when giving special mouth care?

1) Inserting a gauze ball as deep as possible

2) Making a cleaning solution with hydrogen peroxide and water in the proportion of 2:1

3) Tilting the patient's head back if possible because the patient is unconscious

4) Having the patient assume a lateral position to prevent ingestion

5) Measuring the length between the lip and earlobe when determining the size of the oropharyngeal airway

Category	High (27%)	Middle (46%)	Low (27%)	Total	Correct answer rate	Discrimination index	Item-total correlation
No. of correct answers	183	296	160	639	86%	0.12	0.17
No. of wrong answers	17	45	40	102			
Total	200	341	200	741			

Response distribution	No. 1	No. 2	No. 3	No. 4	No. 5	Unanswered	Duplicated	Total
Upper 27%	0	5	2	183	10	0	0	200
Lower 27%	2	13	6	160	19	0	0	200
Total	2	18	8	343	29	0	0	400

Case 8: Questions 17–18

A 53-year-old male patient experienced paralysis on his face 6 months ago and received treatment at a traditional Oriental medicine hospital. However, there was no improvement and he was diagnosed with parotid gland cancer with lung metastasis. It has now been 3 days since he started to receive chemotherapy. The patient says to the nurse, "My stomach churns whenever I see my meals. I vomited up lunch yesterday. I feel poorly and I even have difficulty swallowing."

Job	D2. Nutrition management	Area of learning objectives (category /	Nutritive requirements
Integrated	Checking and analyzing the patient's nutritional		

nursing knowledge	condition	division)	

17. What nursing diagnosis is the first priority for intervention based on the chief complaint of this patient?

1) Risk of injury from a fall caused by low energy

2) Deterioration of body image related to facial muscle paralysis

3) Anxiety related to the occurrence of unexpected disease

4) Not undergoing treatment due to lacking knowledge about the treatment procedure

5) Risk of undernourishment related to nausea/vomiting and impaired swallowing

Category	High (27%)	Middle (46%)	Low (27%)	Total	Correct answer rate	Discrimination index	Item-total correlation
No. of correct answers	200	335	191	726	98%	0.05	0.20
No. of wrong answers	0	6	9	15			
Total	200	341	200	741			

Response distribution	No. 1	No. 2	No. 3	No. 4	No. 5	Unanswered	Duplicated	Total
Upper 27%	0	0	0	0	200	0	0	200
Lower 27%	2	6	0	1	191	0	0	200
Total	2	6	0	1	391	0	0	400

Job	D2. Nutrition management	Area of learning objectives (category / division)	Nutritive requirements
Integrated nursing knowledge	Assessment and management of nutritional issues by disease		

18. Which is an appropriate nursing intervention regarding this patient's situation?

1) Performing mouth care before the patient's intake of food

2) Encouraging the patient to consume high-calorie, fatty foods

3) Providing foods that are warmer than room temperature

4) Tilting the patient's head back in a sitting position in order for him to swallow food well

5) Encouraging him to eat a lot at one time because every meal induces vomiting

Category	High (27%)	Middle (46%)	Low (27%)	Total	Correct answer rate	Discrimination index	Item-total correlation
No. of correct answers	151	200	87	438	59%	0.32	0.26
No. of wrong answers	49	141	113	303			
Total	200	341	200	741			

Response distribution	No. 1	No. 2	No. 3	No. 4	No. 5	Unanswered	Duplicated	Total
Upper 27%	151	34	15	0	0	0	0	200
Lower 27%	87	57	32	16	7	1	0	200
Total	238	91	47	16	7	1	0	400

Case 9: Questions 19–21

A 55-year-old male patient was diagnosed with chronic obstructive pulmonary disease (COPD) and right pneumothorax, and a chest tube drainage (CTD) tube was inserted today. When he lies down, it becomes harder for him to breathe, so he calls the nurse 3 to 4 times during the night. Oxygen was increased from 1 L/min to 2 L/min from a nasal cannula. He just sits on the bed because he cannot sleep soundly due to sticky sputum and a frequent cough.

Job	D5. Provision of sleep and rest	Area of learning objectives	Safety requirements
Integrated nursing	Nursing assessment and interventions related to		

knowledge	sleep requirements	(category / division)	

19. Which is the correct nursing intervention to help this patient sleep?

1) Drinking warm milk before sleep

2) Encouraging discharge of sputum by performing dependent drainage before sleeping

3) Injecting a painkiller 2 hours before sleep to reduce pain

4) Using the light at the patient’s bedside to check the CTD tube

5) Encouraging exercise right before sleep to reduce muscle fatigue

Category	High (27%)	Middle (46%)	Low (27%)	Total	Correct answer rate	Discrimination index	Item-total correlation
No. of correct answers	191	315	172	678	91%	0.10	0.17
No. of wrong answers	9	26	28	63			
Total	200	341	200	741			

Response distribution	No. 1	No. 2	No. 3	No. 4	No. 5	Unanswered	Duplicated	Total
Upper 27%	2	191	4	3	0	0	0	200
Lower 27%	7	172	13	5	3	0	0	200
Total	9	363	17	8	3	0	0	400

Job	E1. Breathing maintenance nursing	Area of learning objectives (category / division)	Oxygenation nursing
Integrated nursing knowledge	Oxygen therapy and evaluating results		

20. What is the FiO_2 of oxygen that this patient currently absorbs?

1) 20% 2) 24% 3) 28% 4) 32% 5) 36%

Category	High (27%)	Middle (46%)	Low (27%)	Total	Correct answer rate	Discrimination index	Item-total correlation
No. of correct answers	122	179	72	373	50%	0.25	0.18
No. of wrong answers	78	162	128	368			
Total	200	341	200	741			

Response distribution	No. 1	No. 2	No. 3	No. 4	No. 5	Unanswered	Duplicated	Total
Upper 27%	27	38	122	10	3	0	0	200
Lower 27%	28	67	72	29	4	0	0	200
Total	55	105	194	39	7	0	0	400

Job	E1. Breathing maintenance nursing	Area of learning objectives (category / division)	Oxygenation nursing
Integrated nursing knowledge	Nursing intervention suitable for the characteristics of respiratory disease		

21. Which is the most effective oxygen supply for this patient?

1) An oxygen tent

2) A Venturi mask

3) A general oxygen mask

4) A partial rebreathing system

5) A non-rebreather oxygen mask with a reservoir bag

Category	High (27%)	Middle (46%)	Low (27%)	Total	Correct answer rate	Discrimination index	Item-total correlation
No. of correct answers	135	169	73	377	51%	0.31	0.25
No. of wrong answers	65	172	127	364			
Total	200	341	200	741			

Response distribution	No. 1	No. 2	No. 3	No. 4	No. 5	Unanswered	Duplicated	Total
Upper 27%	0	135	34	25	6	0	0	200
Lower 27%	3	73	57	30	37	0	0	200
Total	3	208	91	55	43	0	0	400

Case 10: Questions 22–23

A newborn baby who was born at the gestational age of 40 weeks with a birthweight of 3.0 kg has not been eating well for 4 days. Therefore, the baby was admitted into a neonatal intensive care unit (NICU). The color of the baby's eyes and body is yellow and the baby has soft stools. A blood test showed a total bilirubin level of 15 mg/dL.

Job	C1. Monitoring the patient's status	Area of learning objectives	2. Children's growth and development and maintenance and promotion
Integrated nursing	Monitoring the patient's status – Nursing newborn babies with various health problems,		

knowledge	light therapy for high-risk neonates	(category / division)	of health

22. What nursing intervention is the first priority for this newborn baby?

1) Starting light therapy

2) Starting skin nursing

3) Preparing an exchange transfusion

4) Observing its stool

5) Administering saline solution intravenously

Category	High (27%)	Middle (46%)	Low (27%)	Total	Correct answer rate	Discrimination index	Item-total correlation
No. of correct answers	184	307	165	656	89%	0.10	0.15
No. of wrong answers	16	34	35	85			
Total	200	341	200	741			

Response distribution	No. 1	No. 2	No. 3	No. 4	No. 5	Unanswered	Duplicated	Total
Upper 27%	184	2	2	5	7	0	0	200
Lower 27%	165	5	2	12	16	0	0	200
Total	349	7	4	17	23	0	0	400

Job	C1. Monitoring the patient's status	Area of learning objectives (category / division)	2. Children's growth and development and maintenance and promotion of health
Integrated nursing knowledge	Monitoring the patient's status – Nursing newborn babies with various health problems, light therapy for high-risk neonates		

23. This newborn baby's skin color is still yellow and body temperature is warm and variable, at 37.5℃ 24 hours after light therapy was started. The color of the baby's urine is yellow, and its daily intake, excretion, and total bilirubin are 360 cc/day, 200 cc/day, and 14 mg/dL, respectively. What is the most important nursing intervention for this newborn baby?

1) Increasing body temperature through light therapy

2) Provision of significant touch and visual stimuli

3) Sufficient breast-feeding for quick recovery

4) Turning and repositioning every two hours and observing the condition of its skin

5) Provision of water and electrolytes to increase excretion

Category	High (27%)	Middle (46%)	Low (27%)	Total	Correct answer rate	Discrimination index	Item-total correlation
No. of correct answers	93	117	53	263	35%	0.20	0.16
No. of wrong answers	107	224	147	478			
Total	200	341	200	741			

Response distribution	No. 1	No. 2	No. 3	No. 4	No. 5	Unanswered	Duplicated	Total
Upper 27%	32	9	8	93	58	0	0	200
Lower 27%	37	14	26	53	70	0	0	200
Total	69	23	34	146	128	0	0	400

Case 11: Questions 24–25

An 11-month-year infant was admitted to a hospital because the baby showed watery diarrhea 5 times a day, and twice-daily vomiting for 2 days. The test results showed a sodium (NaCl) level of 131.6 mEq/L, a C-reactive protein (CRP) level of 10.8 mg/dL, and the patient sleeps a lot.

Job	E3. Controlling and maintaining	Area of	3. Pediatric health - nursing for children

	metabolism	learning objectives (category / division)	with digestive disorders
Integrated nursing knowledge	Nursing interventions for maintenance of water and electrolyte balance,<br>D4. Nursing interventions for gastrointestinal disorders		

24. Which nursing intervention is the first priority for this infant patient?

1) Providing prescription antibiotics

2) Isolating the baby to prevent infection

3) Performing skin nursing in the anal region

4) Providing albumin by prescription

5) Providing saline solution by prescription

Category	High (27%)	Middle (46%)	Low (27%)	Total	Correct answer rate	Discriminati on index	Item-total correlation
No. of correct answers	112	171	79	362	49%	0.17	0.13
No. of wrong answers	88	170	121	379			
Total	200	341	200	741			

Response distribution	No. 1	No. 2	No. 3	No. 4	No. 5	Unanswered	Duplicated	Total
Upper 27%	48	11	10	19	112	0	0	200
Lower 27%	50	17	17	37	79	0	0	200
Total	98	28	27	56	191	0	0	400

Job	E3. Controlling and maintaining metabolism	Area of learning objectives (category / division)	3. Pediatric health - nursing for children with digestive disorders

Integrated nursing knowledge	Nursing interventions for maintenance of water and electrolyte balance,<br>D4. Nursing interventions for gastrointestinal disorders		

25. Which nursing intervention is appropriate for this infant patient?

1) Fasting

2) Measuring oxygen saturation

3) Performing abdominal ultrasound

4) Measuring intake/excretion

5) Measuring vital signs every hour

Category	High (27%)	Middle (46%)	Low (27%)	Total	Correct answer rate	Discrimination index	Item-total correlation
No. of correct answers	161	261	134	556	75%	0.14	0.12
No. of wrong answers	39	80	66	185			
Total	200	341	200	741			

Response distribution	No. 1	No. 2	No. 3	No. 4	No. 5	Unanswered	Duplicated	Total
Upper 27%	32	0	4	161	3	0	0	200
Lower 27%	42	3	7	134	14	0	0	200
Total	74	3	11	295	17	0	0	400

Case 12: Questions 26–27

A 3-year-old girl was admitted to a hospital with cough, dyspnea, and a high fever of 39°C. Her temperature after hospitalization was measured to be 37.2°C. There are sounds of heavy breathing during inhalation, her breathing rate is 60 breaths/min, and she continues to cough.

Job	E1. Respiration maintenance nursing	Area of learning objectives (category / division)	3. Pediatric health - nursing for children with respiratory disorders
Integrated nursing knowledge	Interpretation of vital signs and taking measures if needed		

26. What is most important nursing intervention for this patient?

1) Observing the patient's body temperature and whether she is experiencing chills

2) Performing chest physiotherapy

3) Monitoring oxygen saturation

4) Providing grape sugar and nutritional supplements

5) Performing endotracheal suction

Category	High (27%)	Middle (46%)	Low (27%)	Total	Correct answer rate	Discrimination index	Item-total correlation
No. of correct answers	154	189	93	436	59%	0.31	0.25
No. of wrong answers	46	152	107	305			
Total	200	341	200	741			

Response distribution	No. 1	No. 2	No. 3	No. 4	No. 5	Unanswered	Duplicated	Total
Upper 27%	20	22	154	1	3	0	0	200
Lower 27%	50	40	93	2	15	0	0	200
Total	70	62	247	3	18	0	0	400

Job	E1. Maintaining respiration	Area of learning objectives (category / division)	3. Pediatric health - nursing for children with respiratory disorders
Integrated nursing knowledge	Chest physiotherapy and evaluation of the results		

27. On the next day, the pediatric patient's cough and phlegm became severe and the rattling sound during breathing and noise heard through the stethoscope became serious. Her body temperature was observed to be 37.0℃ and her breathing rate was 50 breaths/min. Which of the following interventions should be performed for this patient?

1) Education on mask-wearing

2) Monitoring an ECG

3) Administering an oral fever reducer

4) Performing chest physiotherapy continuously

5) Stabilizing the patient by having her assume a supine position

<table>
<tr><th>Category</th><th>High (27%)</th><th>Middle (46%)</th><th>Low (27%)</th><th>Total</th><th>Correct answer rate</th><th>Discrimination index</th><th>Item-total correlation</th></tr>
<tr><td>No. of correct answers</td><td>177</td><td>287</td><td>140</td><td>604</td><td rowspan="3">82%</td><td rowspan="3">0.19</td><td rowspan="3">0.21</td></tr>
<tr><td>No. of wrong answers</td><td>23</td><td>54</td><td>60</td><td>137</td></tr>
<tr><td>Total</td><td>200</td><td>341</td><td>200</td><td>741</td></tr>
</table>

Response distribution	No. 1	No. 2	No. 3	No. 4	No. 5	Unanswered	Duplicated	Total
Upper 27%	6	6	5	177	6	0	0	200
Lower 27%	26	20	6	140	8	0	0	200
Total	32	26	11	317	14	0	0	400

Case 13: Questions 28–30

A 11-year-old female patient showed albuminuria in a physical examination for school in June 2012, and edema around the eyes and anasarca occurred. She was admitted to a hospital, diagnosed with nephrotic syndrome (NS), and has been taking a steroid since hospitalization. Recently, she visited a hospital with edema around her eyes and on her face, weight loss of 3 kg, reduction of urine output, abdominal pain, albuminuria, and hematuria as her chief complaints. The laboratory findings were as follows: albuminuria (+++), blood urea nitrogen (BUN) of 70 mg/dL,

uric acid of 12.8 mg/dL, UA microalbumin +++, and many red blood cells per HPF. Her serum albumin level, cholesterol level, and 24-hour urinary protein were 2.5 g/dL, 350 mg/dL, and 1.8 g/24 h, respectively.

Job	E3. Control and maintenance of metabolism	Area of learning objectives (category / division)	3. Pediatric health - nursing for children with genetic and elimination disorders
Integrated nursing knowledge	Nursing interventions for maintenance of water-electrolyte balance, assessing body fluid-electrolyte imbalances and intervening		

28. What should be done first with this patient?

1) Check her pain level 2) Check her state of consciousness 3) Check her blood pressure every hour

4) Check oxygen saturation 5) Check intake and output

Category	High (27%)	Middle (46%)	Low (27%)	Total	Correct answer rate	Discrimination index	Item-total correlation
No. of correct answers	158	262	141	561	76%	0.09	0.10
No. of wrong answers	42	79	59	180			
Total	200	341	200	741			

Response distribution	No. 1	No. 2	No. 3	No. 4	No. 5	Unanswered	Duplicated	Total
Upper 27%	14	19	8	1	158	0	0	200
Lower 27%	15	30	12	2	141	0	0	200
Total	29	49	20	3	299	0	0	400

Job	E3. Controlling and maintaining metabolism	Area of learning objectives (category / division)	3. Pediatric health - nursing for children with genetic and elimination disorders
Integrated nursing knowledge	Nursing interventions for maintenance of water–electrolyte balance, assessing symptoms and signs of body fluid-electrolyte imbalance and intervening		

29. What is the most important nursing intervention for this patient?

1) Encouraging oral water intake

2) Injecting fluids by prescription

3) Measuring vital signs every hour

4) Injecting albumin by prescription

5) Having the patient eat delicious food, as she has a poor appetite

Category	High (27%)	Middle (46%)	Low (27%)	Total	Correct answer rate	Discrimination index	Item-total correlation
No. of correct answers	131	161	72	364	49%	0.30	0.22
No. of wrong answers	69	180	128	377			
Total	200	341	200	741			

Response distribution	No. 1	No. 2	No. 3	No. 4	No. 5	Unanswered	Duplicated	Total
Upper 27%	10	28	28	131	3	0	0	200
Lower 27%	25	68	29	72	6	0	0	200
Total	35	96	57	203	9	0	0	400

Job	H1. Educating	Area of learning objectives (category / division)	3. Pediatric health - nursing for children with genetic and elimination disorders
Integrated nursing knowledge	Nursing interventions for maintenance of water-electrolyte balance, assessing symptoms and signs of body fluid-electrolyte imbalance and intervening		

30. Which of the following is an appropriate form of patient education?

1) Encouraging infection prevention

2) Encouraging water intake

3) Encouraging salt intake

4) Encouraging potassium intake

5) Encouraging fat intake

Category	High (27%)	Middle (46%)	Low (27%)	Total	Correct answer rate	Discrimination index	Item-total correlation
No. of correct answers	106	113	46	265	36%	0.30	0.27
No. of wrong answers	94	228	154	476			
Total	200	341	200	741			

Response distribution	No. 1	No. 2	No. 3	No. 4	No. 5	Unanswered	Duplicated	Total
Upper 27%	106	46	9	31	8	0	0	200
Lower 27%	46	94	9	47	4	0	0	200
Total	152	140	18	78	12	0	0	400

Case 14: Questions 31–32

A 32-year-old multigravida at 30 weeks of pregnancy visited an emergency room with vaginal bleeding. Her health status when she visited the emergency room was as follows.

· V/S: T 36.7°C, P 70 beats per minute, R 20 breaths per minute, BP 120/70 mm Hg

· Vaginal bleeding: Vaginal spotting started 4 hours ago and one sanitary pad has been soaked.

· Uterine contractions: None

· Fetal heart rate: 138 beats/min

· Results of blood test: hemoglobin (Hb) 8.9 g/dL, hematocrit (Hct) 25.2%

Job	C5. Prenatal/intranatal/postnatal nursing<br>H3. Checking health risk factors	Area of learning objectives (category / division)	Pregnant women-hemorrhagic health problems
Integrated nursing knowledge	Explaining treatment methods to high-risk pregnant woman and nursing		

31. Which event is likely to be taking place in this pregnant woman?

1) Miscarriage 2) Hydatid mole 3) Placenta previa 4) Abruptio placentae 5) Cervical incompetency

Category	High (27%)	Middle (46%)	Low (27%)	Total	Correct answer rate	Discrimination index	Item-total correlation
No. of correct answers	74	72	35	181	27%	0.20	0.20
No. of wrong answers	126	269	165	560			
Total	200	341	200	741			

Response distribution	No. 1	No. 2	No. 3	No. 4	No. 5	Unanswered	Duplicated	Total
Upper 27%	6	5	74	74	41	0	0	200
Lower 27%	3	9	35	125	28	0	0	200
Total	9	14	109	199	69	0	0	400

Job	C5. Prenatal/intranatal/postnatal nursing<br>C1. Monitoring the patient's status	Area of learning objectives (category / division)	Pregnant women – hemorrhagic health problems
Integrated nursing knowledge	Explaining treatment methods to high-risk pregnant and nursing women		

32. Which of the following is an appropriate, high-priority nursing intervention?

1) Having the patient rest completely

2) Checking whether there is amniorhexis

3) Securing a vein in preparation for blood transfusion

4) Checking urine volume by inserting a Foley catheter

5) Supplying oxygen at 3L/min through an oxygen mask

Category	High (27%)	Middle (46%)	Low (27%)	Total	Correct answer rate	Discrimination index	Item-total correlation
No. of correct answers	115	167	73	355	48%	0.21	0.19
No. of wrong answers	85	174	127	386			
Total	200	341	200	741			

Response distribution	No. 1	No. 2	No. 3	No. 4	No. 5	Unanswered	Duplicated	Total
Upper 27%	115	61	20	0	4	0	0	200
Lower 27%	73	87	29	5	6	0	0	200
Total	188	148	49	5	10	0	0	400

Case 15: Questions 33–34

Last night, a 29-year-old primigravida at 41 weeks and 3 days of pregnancy was admitted to a delivery room for induced delivery.

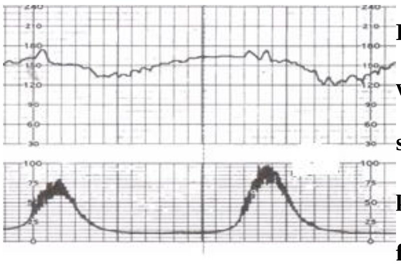

Beginning at 09:00 this morning, when an intravenous injection of Hartman solution mixed with oxytocin was started, the injection volume was increased continuously. Currently, the delivery status examination shows dilatation of 2 cm, cervical effacement of 70%, and presenting part descent of −3. The graph below was observed during electronic fetal monitoring (EFM).

Job	C5. Prenatal/intranatal/postnatal nursing<br>C1. Monitoring the patient's status	Area of learning objectives (category / division)	5. Pregnant women - fetal health assessment and nursing<br>6. Parturition - alternative delivery
Integrated nursing knowledge	Nursing according to high-risk delivery type		

33. What can be identified through the above electronic fetal monitoring graph?

1) Weak pain 2) Fetal head pressure 3) Cord blood pressure 4) Active fetal movement 5) Uteroplacental circulation insufficiency

Category	High (27%)	Middle (46%)	Low (27%)	Total	Correct answer rate	Discrimination index	Item-total correlation
No. of correct answers	80	78	34	192	26%	0.23	0.18
No. of wrong answers	120	263	166	549			
Total	200	341	200	741			

Response distribution	No. 1	No. 2	No. 3	No. 4	No. 5	Unanswered	Duplicated	Total
Upper 27%	6	37	68	9	80	0	0	200
Lower 27%	16	51	74	25	34	0	0	200
Total	22	88	142	34	114	0	0	400

Job	C5. Prenatal/intranatal/postnatal nursing<br>C1. Monitoring the patient's status	Area of learning objectives (category / division)	5. Pregnant women - fetal health assessment and nursing<br>6. Parturition - alternative delivery
Integrated nursing knowledge	Nursing according to high-risk delivery type		

34. Which nursing intervention should be performed by the nurse as a top priority?

1) Having the patient assume a semirecumbent position

2) Injecting oxygen

3) Raising the patient's legs

4) Increasing the volume of the intravenous injection

5) Stopping the oxytocin injection

Category	High (27%)	Middle (46%)	Low (27%)	Total	Correct answer rate	Discrimination index	Item-total correlation
No. of correct answers	82	94	37	213	29%	0.23	0.17
No. of wrong answers	118	247	163	528			
Total	200	341	200	741			

Response distribution	No. 1	No. 2	No. 3	No. 4	No. 5	Unanswered	Duplicated	Total
Upper 27%	98	16	2	2	82	0	0	200
Lower 27%	98	37	12	15	37	1	0	200
Total	196	53	14	17	119	1	0	400

Case 16: Questions 35–36

A **30-year-old primigravida at 34 weeks and 5 days pregnant visited a gynecology outpatient clinic with severe headache as a chief complaint. Her blood pressure was 160/110 mm Hg and the pitting edema in her arm was +3. She had gained 2 kg of weight over the last week and albuminuria was confirmed. The non-stress test (NST) was reactive. She was admitted to the hospital and intravenous $MgSO_4$ was prescribed.**

Job	C5. Prenatal/intranatal/postnatal nursing<br>H3. Checking health risk factors	Area of learning	5. Pregnant women

Integrated nursing knowledge	Explaining treatment methods to high-risk pregnant and nursing women	objectives (category / division)	9) Hypertensive health problems

35. Which of the following would provide an appropriate environment in the patient room where this pregnant woman is staying?

1) Turning the lights up

2) Leaving the bed rail down

3) Keeping the room quiet

4) Permitting free access of visitors

5) Providing the patient with a multiple-patient room

Category	High (27%)	Middle (46%)	Low (27%)	Total	Correct answer rate	Discrimination index	Item-total correlation
No. of correct answers	200	340	190	730	99%	0.05	0.23
No. of wrong answers	0	1	10	11			
Total	200	341	200	741			

Response distribution	No. 1	No. 2	No. 3	No. 4	No. 5	Unanswered	Duplicated	Total
Upper 27%	0	0	200	0	0	0	0	200
Lower 27%	3	3	190	3	1	0	0	200
Total	3	3	390	3	1	0	0	400

Job	C5. Prenatal/intranatal/postnatal nursing<br>C1. Monitoring the patient's status	Area of learning objectives (category / division)	5. Pregnant women<br>9) Hypertensive health problems
Integrated nursing knowledge	Explaining treatment methods to high-risk pregnant and nursing women		

36. What should the nurse watch for carefully during the intravenous administration of $MgSO_4$?

1) Increased blood pressure

2) Increased pulse rate

3) Decreased breathing rate

4) Increased amount of urine

5) Increased deep tendon reflex (DTR)

Category	High (27%)	Middle (46%)	Low (27%)	Total	Correct answer rate	Discrimination index	Item-total correlation
No. of correct answers	86	98	41	225	30%	0.23	0.20
No. of wrong answers	114	243	159	516			
Total	200	341	200	741			

Response distribution	No. 1	No. 2	No. 3	No. 4	No. 5	Unanswered	Duplicated	Total
Upper 27%	27	23	86	8	56	0	0	200
Lower 27%	67	43	41	16	33	0	0	200
Total	94	66	127	24	89	0	0	400

Case 17: Questions 37–40

A patient was admitted to a hospital with one of the following statements as a chief complaint: "The National Intelligence Service is watching me because I am an important figure," or "Somebody comes inside my head and steals my ideas."

Job	G1. Supporting patients and their family	Area of learning objectives (category / division)	Nursing - schizophrenia and other psychotic disorders
Integrated nursing knowledge	Nursing for schizophrenic patients and their family		

37. Which of the following is a correct nursing intervention for the chief complaint of the patient?

1) Supporting the complaint of the patient and accepting the patient's emotions

2) Reassessing which behaviors caused by hallucinations are problematic

3) Telling right from wrong based on the behavioral characteristics of the patient

4) Approaching the patient based on everyday problems rather than logical solutions

5) Reassuring the patient by saying that nothing will happen in the hospital

Category	High (27%)	Middle (46%)	Low (27%)	Total	Correct answer rate	Discrimination index	Item-total correlation
No. of correct answers	80	118	59	257	35%	0.11	0.09
No. of wrong answers	120	223	141	484			
Total	200	341	200	741			

Response distribution	No. 1	No. 2	No. 3	No. 4	No. 5	Unanswered	Duplicated	Total
Upper 27%	53	36	5	80	26	0	0	200
Lower 27%	44	49	13	59	35	0	0	200
Total	97	85	18	139	61	0	0	400

Job	G1. Supporting patients and their family	Area of learning objectives (category / division)	Nursing - schizophrenia and other psychotic disorders
Integrated nursing knowledge	Therapeutic communication techniques		

38. “This is my third hospital admission. I’ve been admitted to hospitals since I was 20, so I’ve spent my 20s in hospitals. What can I do now?” The above patient is worried, with a grim face. In this situation, which of the following would be an effective communication technique that the nurse could use?

1) “What are you talking about? You are just starting out in life. Life is so long.”

2) “You must be worried because you are admitted to hospitals over and over. What do you want to do?”

3) “Just think about your parents. They are doing their best.”

4) “You must feel this way after seeing patients here who go and come repeatedly, right? Please participate in therapy activities more actively. They will guide you.”

5) “You can do well. You will be able to get a job. Let me introduce you to a social rehabilitation center that provides counseling and occupational rehabilitation. I hear that most patients become employed through the center.”

Category	High (27%)	Middle (46%)	Low (27%)	Total	Correct answer rate	Discrimination index	Item-total correlation
No. of correct answers	187	279	137	603	81%	0.25	0.29
No. of wrong answers	13	62	63	138			
Total	200	341	200	741			

Response distribution	No. 1	No. 2	No. 3	No. 4	No. 5	Unanswered	Duplicated	Total
Upper 27%	0	187	0	6	7	0	0	200
Lower 27%	5	137	3	34	21	0	0	200
Total	5	324	3	40	28	0	0	400

Job	G1. Supporting patients and their family	Area of learning objectives (category / division)	Nursing - schizophrenia and other psychotic disorders
Integrated nursing knowledge	Therapeutic communication techniques		

39. The family members of the patient say that it is hard for them to see the patient because of the patient's major symptoms. Which response would be correct in this situation?

1) "Don't worry, the patient is in a psychiatric ward. She will get better once she attends the program according to the treatment plan."

2) "It's hard. Yes, you must be upset. Are you afraid her stay in the hospital will become longer?"

3) "That is the most difficult symptom to treat now. The patient will probably become stable after about 3 months of care in the hospital."

4) "It seems that she did not take her medications after leaving the hospital. The disease may recur unless she takes the medications. Treatment with medication yield better results."

5) "Maybe the doctor in charge explained this to you: to control her neurotransmitters, the patient should keep taking the medicine that she is taking now. That's how a condition like delirium is controlled."

Category	High (27%)	Middle (46%)	Low (27%)	Total	Correct answer rate	Discrimination index	Item-total correlation
No. of correct answers	162	220	85	467	63%	0.39	0.31
No. of wrong answers	38	121	115	274			
Total	200	341	200	741			

Response distribution	No. 1	No. 2	No. 3	No. 4	No. 5	Unanswered	Duplicated	Total
Upper 27%	12	162	1	7	18	0	0	200
Lower 27%	45	85	4	28	38	0	0	200
Total	57	247	5	35	56	0	0	400

Job	G5. Crisis management	Area of learning	Nursing - schizophrenia and other

Integrated nursing knowledge	Assessment of clues about suicide	objectives (category / division)	psychotic disorders

40. The above patient says on the second week of hospitalization, “The National Intelligence Service is finally attempting to take my life, because I am an important person. They are in my head wherever I go. I think I'd rather die. Thank you for everything you've done for me.” She expresses gratitude to other patients as well. What is a correct nursing intervention in this situation?

1) Assessing her for suicidal ideation

2) Determining a regular time to observe her

3) Allowing the patient to take a walk as a diversion

4) Moving the patient to a solitary room for intensive observation

5) Increasing activity programs for various treatments

Category	High (27%)	Middle (46%)	Low (27%)	Total	Correct answer rate	Discrimination index	Item-total correlation
No. of correct answers	190	306	154	650	88%	0.18	0.24
No. of wrong answers	10	35	46	91			
Total	200	341	200	741			

Response distribution	No. 1	No. 2	No. 3	No. 4	No. 5	Unanswered	Duplicated	Total
Upper 27%	190	7	0	2	1	0	0	200
Lower 27%	154	26	2	14	4	0	0	200
Total	344	33	2	16	5	0	0	400

Case 18: Questions 41–43

A patient wore thick and exaggerated make-up inappropriately and a colorful floral-patterned dress when she was admitted to a hospital. However, she also had tangled hair and dirty fingernails. As soon as she entered the ward, she went around at a rapid pace and shook hands with unfamiliar patients. She even gave them nicknames and called

them by those nicknames. Her chief complaints were statements such as, "I am too busy to eat meals. I don't have time to eat meals because I am so excited about my ideas. I just cannot eat meals because I don't want to waste my time. Why are they so loud?" She told others that it was problematic that her family admitted her to a hospital despite having no reason for admission. According to her family, she lost 8 kg of weight over the last month as she went around saying she was busy. Even though she was unemployed, she bought 10 newly released cell phones and handed out them to her friends one week before her hospitalization. The family bore the burden for the price of those cellphones and now say that they are extremely exhausted and hate the very sight of her.

Job	C1. Monitoring the patient's status	Area of learning objectives (category / division)	Nursing - mood-related disorders
Integrated nursing knowledge	Assessment of patient's mental state		

41. What question should the nurse ask the above patient to assess her major mental state?

1) "How are you feeling today?"

2) "Where are we?"

3) "What are you thinking about?"

4) "Please subtract 5 from 100 five times."

5) "Please say '*Sam-cheol-li-gang-san*' backward."

Category	High (27%)	Middle (46%)	Low (27%)	Total	Correct answer rate	Discrimination index	Item-total correlation
No. of correct answers	151	222	87	460	62%	0.32	0.29
No. of wrong answers	49	119	113	281			
Total	200	341	200	741			

Response distribution	No. 1	No. 2	No. 3	No. 4	No. 5	Unanswered	Duplicated	Total
Upper 27%	151	12	25	6	6	0	0	200
Lower 27%	87	26	69	12	6	0	0	200

Total	238	38	94	18	12	0	0	400

Job	G1. Supporting patients and their family<br>D2. Nutrition management	Area of learning objectives (category / division)	Nursing - mood-related disorders
Integrated nursing knowledge	Checking and analyzing the patient's nutritional condition		

42. What is a high-priority nursing diagnosis regarding this patient's behavioral characteristics?

1) Fatigue

2) Nutritional imbalance

3) Sleeping difficulties

4) Social interaction

5) Linguistic communication disorder

Category	High (27%)	Middle (46%)	Low (27%)	Total	Correct answer rate	Discrimination index	Item-total correlation
No. of correct answers	179	253	116	548	74%	0.32	0.29
No. of wrong answers	21	88	84	193			
Total	200	341	200	741			

Response distribution	No. 1	No. 2	No. 3	No. 4	No. 5	Unanswered	Duplicated	Total
Upper 27%	9	179	3	9	0	0	0	200
Lower 27%	23	116	4	55	2	0	0	200
Total	32	295	7	64	2	0	0	400

Job	F1. Administering drugs	Area of learning objectives (category / division)	Nursing - mood-related disorders
Integrated nursing knowledge	Administration of medicine according to a systems-based approach to disease and medication-related nursing		

43. Lithium was prescribed to the patient and she is experiencing diarrhea and vomiting. Which nursing intervention should be carried out as the highest priority?

1) Having the patient take bed rest

2) Starting fluid therapy

3) Stopping the patient from attending therapy activities and having her rest

4) Suspending the medication and discussing the patient's symptoms with the doctor in charge.

5) Having the patient drink tepid water frequently to maintain hydration.

Category	High (27%)	Middle (46%)	Low (27%)	Total	Correct answer rate	Discrimination index	Item-total correlation
No. of correct answers	156	246	101	503	68%	0.28	0.21
No. of wrong answers	44	95	99	238			
Total	200	341	200	741			

Response distribution	No. 1	No. 2	No. 3	No. 4	No. 5	Unanswered	Duplicated	Total
Upper 27%	2	17	2	156	23	0	0	200
Lower 27%	4	31	14	101	50	0	0	200
Total	6	48	16	257	73	0	0	400

Case 19: Questions 44–46

A Community health nurse in charge of case management for the Dream Start program, including "A," a male child from a socially disadvantaged class, assessed A's family health—including A's grandparents—as follows after visiting their family, which receives basic living security. A's father has class 1 mental retardation and A's mother is a Philippine marriage immigrant who can speak only simple Korean. A is slow developing linguistic and mathematical skills compared to his peers. Therefore, mental retardation is suspected.

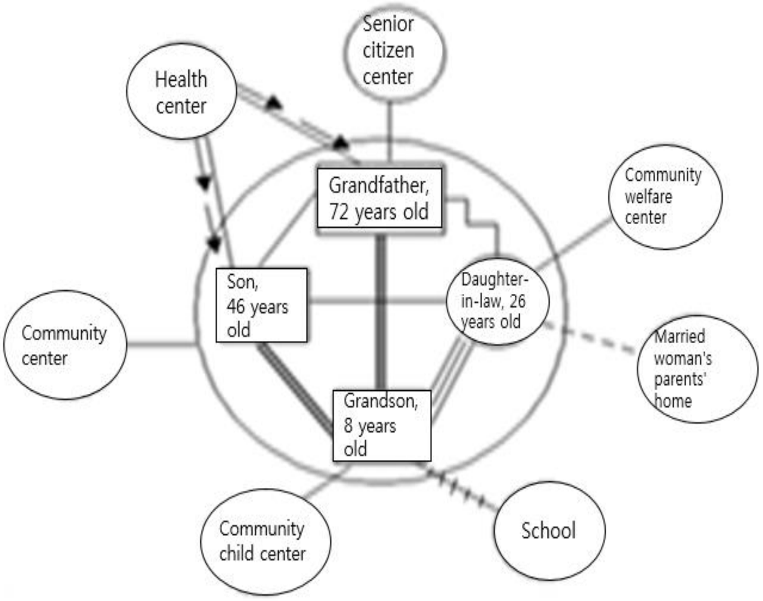

Job	A10. Utilizing health care service resources<br>A9. Enhancement of professional capabilities	Area of learning objectives (category / division)	Family nursing
Integrated nursing knowledge	Selection of various resources and media that are appropriate for subjects of nursing / effective communication and putting words into behavior / primary care-based health nursing and participation in health promotion activities		

44. Which of the following is an appropriate interpretation of the family health assessment tool?

1) The daughter-in-law and grandson have a conflicting relationship.

2) The daughter-in-law engages with her parents' home actively.

3) The grandson does not engage much with the community child center.

4) The grandfather is estranged from his daughter-in-law.

5) The grandson experiences stress due to school.

Category	High (27%)	Middle (46%)	Low (27%)	Total	Correct answer rate	Discrimination index	Item-total correlation
No. of correct answers	151	241	113	505	68%	0.19	0.16
No. of wrong answers	49	100	87	236			

Total	200	341	200	741			

Response distribution	No. 1	No. 2	No. 3	No. 4	No. 5	Unanswered	Duplicated	Total
Upper 27%	0	0	8	41	151	0	0	200
Lower 27%	2	1	14	70	113	0	0	200
Total	2	1	22	111	264	0	0	400

Job	A10. Utilizing health care service resources<br>A9. Enhancement of professional capabilities	Area of learning objectives (category / division)	Family nursing
Integrated nursing knowledge	Selection of various resources and media that are appropriate for subjects of nursing / effective communication and putting words into behavior / primary care-based health nursing and participation in health promotion activities		

45. Which of the following is appropriate as a development task across family life cycle stages for the above family?

1) The parents should learn their roles and functions as parents.

2) The family needs rules and regulation internally.

3) The parents should develop a closer relationship by establishing a family plan.

4) The family should acquire techniques to develop a new relationship with the child.

5) The most important developmental task of the child in this period is initiative.

Category	High (27%)	Middle (46%)	Low (27%)	Total	Correct answer rate	Discrimination index	Item-total correlation
No. of correct answers	56	82	43	181	24%	0.07	0.06
No. of wrong answers	144	259	157	560			
Total	200	341	200	741			

Response distribution	No. 1	No. 2	No. 3	No. 4	No. 5	Unanswered	Duplicated	Total
Upper 27%	70	56	7	21	46	0	0	200
Lower 27%	74	43	19	30	33	1	0	200
Total	144	99	26	51	79	1	0	400

Job	A10. Utilizing health care service resources<br>A9. Enhancement of professional capabilities	Area of learning objectives (category / division)	Family nursing
Integrated nursing knowledge	Selection of various resources and media that are appropriate for subjects of nursing / effective communication and putting words into behavior / primary care-based health nursing and participation in health promotion activities		

46. Which of the following is an appropriate nursing diagnosis for A's family?

1) Communication disorders 2) Inappropriate life pattern

3) Inappropriate power structure 4) Inappropriate disease management

5) Inefficient financial management capabilities

Category	High (27%)	Middle (46%)	Low (27%)	Total	Correct answer rate	Discrimination index	Item-total correlation
No. of correct answers	157	241	123	521	70%	0.17	0.17
No. of wrong answers	43	100	77	220			
Total	200	341	200	741			

Response distribution	No. 1	No. 2	No. 3	No. 4	No. 5	Unanswered	Duplicated	Total
Upper 27%	157	18	4	16	5	0	0	200
Lower 27%	123	49	13	5	10	0	0	200
Total	280	67	17	21	15	0	0	400

Case 20: Questions 47–48

A client of a nurse home visiting program was introduced by a senior nurse to a new nurse who was assigned to a health center 3 days ago as follows: "This client is a 75-year-old lady who lives alone and recently moved to our region. She is about to have her first session. She is a recipient of basic living subsidies and has hypertension, diabetes, and mobility difficulties.

Job	A10. Utilizing health care service resources<br>H2. Understanding an individual's health status	Area of learning objectives (category / division)	Nursing of population groups by health problems
Integrated job knowledge	Multidimensional approach plan for health promotion and reduction of danger risks / content analysis of the assessment of health-threatening factors in individuals, families, communities, and population groups		

47. Through what channels can a new nurse infer that the senior nurse primarily obtained the underlined information on the patient?

1) Through hospitals or clinics in the region

2) Through the police station in the region

3) Through the national health insurance service

4) Through the chairman of the senior citizen center in the region

5) Through the integrated case management team in the *gu*-level office in the region

Category	High (27%)	Middle (46%)	Low (27%)	Total	Correct answer rate	Discrimination index	Item-total correlation
No. of correct answers	166	254	123	543	73%	0.22	0.20
No. of wrong answers	34	87	77	198			
Total	200	341	200	741			

Response distribution	No. 1	No. 2	No. 3	No. 4	No. 5	Unanswered	Duplicated	Total
Upper 27%	9	0	25	0	166	0	0	200
Lower 27%	14	1	56	6	123	0	0	200

Total	23	1	81	6	289	0	0	400

Job	A10. Utilizing health care service resources<br>H2. Understanding an individual's health status	Area of learning objectives (category / division)	Nursing of population groups by health problems
Integrated nursing knowledge	Multidimensional approach plan for health promotion and reduction of danger risks / content analysis of the assessment of health-threatening factors in individuals, families, communities, and population groups		

48. A client was introduced to a new nurse. The nurse visited the client at home at about 10:00 the next morning. The client's blood pressure was 170/110 mm Hg and blood glucose was 77 mg/dL. The subject correctly responded to questions about his/her name and age, but did not know the date. What should the nurse do as a first priority?

1) Have the subject rest while sitting and measure his or her blood pressure 5 minutes later. 2) Prepare sugar water and have the subject drink it to prevent hypoglycemic shock.

3) Ask if everything is all right for the subject and then decide what to do next.

4) Call 119 as soon as possible to prepare for the possibility of cerebral hemorrhage.

5) Persuade the subject to visit a nearby hospital for treatment because both blood pressure and blood glucose were at a dangerous level.

Category	High (27%)	Middle (46%)	Low (27%)	Total	Correct answer rate	Discrimination index	Item- total correlation
No. of correct answers	11	19	9	39	5%	0.01	0.00
No. of wrong answers	189	322	191	702			
Total	200	341	200	741			

Response distribution	No. 1	No. 2	No. 3	No. 4	No. 5	Unanswered	Duplicated	Total
Upper 27%	109	75	11	0	5	0	0	200

Lower 27%	101	63	9	5	22	0	0	200
Total	210	138	20	5	27	0	0	400

Case 21: Questions 49–50

A health center in a region where manufacturing is widespread found that the local residents had a high-risk drinking rate of 11.7% in 2008. However, the greatest increase in drinking was found in 2015, with a rate of 25.8%, up by 14.1%. The health center, therefore, wants to establish a regional health care plan that suits the situation of the region based on these basic data.

Job	A10. Utilizing health care service resources<br>A7. Participating in the improvement of nursing quality	Area of learning objectives (category / division)	Community health nursing administration
Integrated nursing knowledge	Selection of various resources and media that are appropriate for the subject of nursing / working to improve nursing quality		

49. What of the following is the highest-priority role that a health nurse of this health center should play?

1) Assessing the health requirements of local community residents

2) Establishing and executing the plan developed by the local community health investigation

3) Deciding the priorities of overall local health care policy

4) Suggesting a nursing diagnosis according to the results of the local community health investigation

5) Suggesting an integrated health promotion project according to the results of the local community health investigation.

Category	High (27%)	Middle (46%)	Low (27%)	Total	Correct answer rate	Discrimination index	Item-total correlation
No. of correct answers	25	32	14	71	10%	0.06	0.05
No. of wrong answers	175	309	186	670			
Total	200	341	200	741			

Response distribution	No. 1	No. 2	No. 3	No. 4	No. 5	Unanswered	Duplicated	Total
Upper 27%	121	14	19	21	25	0	0	200
Lower 27%	106	34	22	24	14	0	0	200
Total	227	48	41	45	39	0	0	400

Job	A10. Utilizing health care service resources<br>A7. Participating in the improvement of nursing quality	Area of learning objectives (category / division)	Community health nursing administration
Integrated nursing knowledge	Selection of various resources and media that are appropriate for the subject of nursing / working to improve nursing quality		

50. Which is an appropriate explanation of the regional health care plan of this health center according to the above basic data?

1) A yearly detailed execution plan is included.

2) The plan is established based on the enforcement of regulations of the National Health Promotion Act.

3) The plan is established every 4 years in accordance with the situation of the region.

4) The head of the health center is installed at the *si/gun/gu* level.

5) Mayors and governors have developed a method for balanced regional development.

Category	High (27%)	Middle (46%)	Low (27%)	Total	Correct answer rate	Discrimination index	Item-total correlation
No. of correct answers	58	96	45	199	27%	0.07	0.09
No. of wrong answers	142	245	155	542			
Total	200	341	200	741			

Response distribution	No. 1	No. 2	No. 3	No. 4	No. 5	Unanswered	Duplicated	Total
Upper 27%	64	53	58	14	11	0	0	200

Lower 27%	54	64	45	23	14	0	0	200
Total	118	117	103	37	25	0	0	400
